# Economic analysis of coronary artery bypass grafting with minimal versus conventional extracorporeal circulation

**DOI:** 10.1186/1749-8090-8-S1-O166

**Published:** 2013-09-11

**Authors:** K Anastasiadis, V Fragoulakis, P Antonitsis, N Maniadakis

**Affiliations:** 1 Department of Cardiothoracic Surgery, AHEPA University Hospital, Thessaloniki, Greece; 2 Department of Health Services Organization & Management, National School of Public Health, Athens, Greece

## Background

This study aims to develop a methodological framework for the comparative economic evaluation between Minimal Extracorporeal Circulation (MECC) versus conventional Extracorporeal Circulation (CECC) in patients undergoing coronary artery bypass grafting (CABG) in different healthcare systems. In addition, the scope is to evaluate the cost-effectiveness ratio of alternative comparators in the healthcare setting of Greece, Germany, the Netherlands and Switzerland.

## Methods

The effectiveness data utilized were derived from a recent meta-analysis which incorporated 24 randomized clinical trials. Total therapy cost per patient reflects all resources expensed in delivery of therapy and the management of any adverse events, including drugs, diagnostics tests, materials, devices, blood units, the utilization of operating theaters, intensive care units and wards. Perioperative mortality was used as the primary health outcome to estimate life years gained in treatment arms. Bias-corrected uncertainty intervals were calculated using the percentile method of non-parametric Monte-Carlo simulation.

## Results

The MECC circuit was more expensive than CECC, with a difference ranging from €180 to €600 depending on the country. However, in terms of total therapy cost per patient the comparison favoured MECC in all countries. Specifically it was associated with a reduction of €789 in Greece, €511 in Germany, €2,343 in the Netherlands and €1,141 in Switzerland. In terms of effectiveness, the total life-years gained were slightly higher in favor of MECC.

## Conclusions

Surgery with MECC may be dominant (lower cost and higher effectiveness) compared to CECC in coronary revascularization procedures and therefore it represents an attractive new option relative to conventional extracorporeal circulation for CABG.

